# Enaminones as Building Blocks in Heterocyclic Syntheses: Reinvestigating the Product Structures of Enaminones with Malononitrile. A Novel Route to 6-Substituted-3-Oxo-2,3-Dihydropyridazine-4-Carboxylic Acids

**DOI:** 10.3390/molecules14010068

**Published:** 2008-12-29

**Authors:** Abdul-aziz Alnajjar, Mervat Mohammed Abdelkhalik, Amal Al-Enezi, Mohamed Hilmy Elnagdi

**Affiliations:** 1 Applied Science Department, College of Technological Studies, Public Authority for Applied Education and Training, P. O. Box 42325 Safat, 70654, Kuwait; 2 Chemistry Department, Kuwait University, P.O. Box 5969 Safat, 13060, Kuwait

**Keywords:** Enaminones, 2-Cyano-5-(dimethylamino)-5-arylpenta-2,4-dienamide, DEPT, Experiments, X-ray crystal structure.

## Abstract

The reported structures of reaction products of enaminones with malononitrile in ethanolic piperidine are revised. A novel route to 2,3-dihydropyridazine-4-carboxylic acids **4a-c*** via* reactions of 2-cyano-5-(dimethylamino)-5-arylpenta-2,4-dienamides **8a-c** with nitrous acid or with benzenediazonium chloride is reported. Compounds **8a-c** are converted to 1,2-dihydropyridine-3-carboxylic acid and 1,2-dihydropyridine-3-carbonitrile derivatives upon reflux in EtOH/ HCl and in AcOH.

## Introduction

**Scheme 1:**
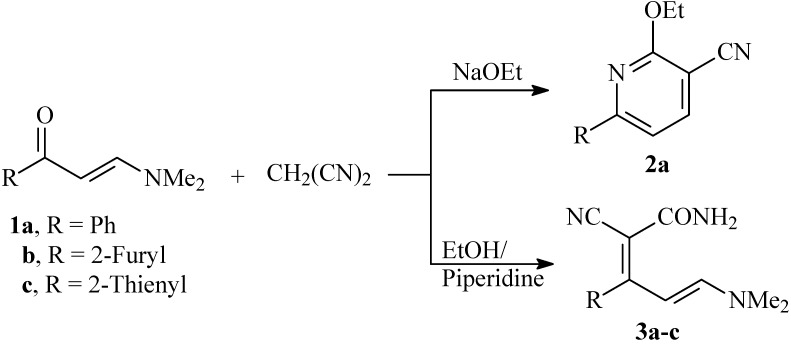
Reported structures for the products of reaction of enaminones **1a-c** and malononitrile.

Enaminones are polydentate reagents that have been utilized extensively in this decade as building blocks in organic synthesis [1,2,3,4,5,6]. In previous work at our laboratories, we reported several efficient routes to polyfunctionally substituted heterocycles utilizing enaminones as starting materials [7,8,9,10,11]. We have also reported that the reaction of **1a** with malononitrile in ethanolic sodium ethoxide afforded **2a** in good yield [12], while reacting **1a-c** with malononitrile in ethanolic piperidine was believed to afford **3a-c** [13] (cf. Scheme 1). In continuation to this work, the chemical reactivity of the products believed to be **3a-c** was reinvestigated. The work has led us to revise the initially proposed structures of these products.

## Results and Discussion

The reaction of **1a-c** with malononitrile in ethanolic piperidine afforded products of molecular formulae corresponding to the formation of 1:1 adducts. As reported earlier [13], the reaction products showed in the ^1^H-NMR spectrum, in addition of the dimethylamino moiety, two olefinic proton doublets at δ_H_ = ca. 5.77 and 7.23 ppm with *J* = 13 Hz, which fits well with the previously assumed initial 1,2-addition of malononitrile at the carbonyl moiety. Subsequent water elimination and hydrolysis of one of the cyano groups into an amide yielded **3a-c. **However, treating these reaction products with sodium nitrite in EtOH/HCl in presence of sodium acetate affords products for which structures **4a-c** are assigned, based on X-ray crystal structure determination [14]. Although the described conditions may not normally lead to hydrolysis of nitriles, however a ready hydrolysis in this case may be prompted by the stabilization of products by potential hydrogen bonding and high reactivity of the nitrile group as part of a π-deficient system. Quite unexpectedly, coupling the products, obtained from the reaction of malononitrile with enaminones **1a-c,** with benzenediazonium chloride in dioxane/AcONa resulted in the formation of the same products **4a-c,** in good yields.

**Figure 1:**
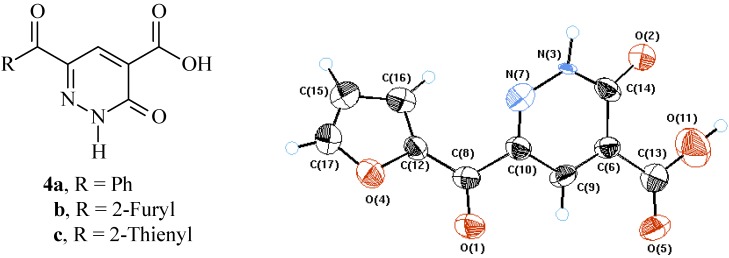
X-Ray crystal structure of **4b**.

There is indication of extensive delocalization of N-1 lone-pair at carbonyl carbon. Thus N-3 bond angles are more like those of sp^2^ nitrogen, while those of N-7-C10-C8 are more like sp^3^ carbon (cf. Figure 1 and Table 1).

**Table 1:** Selected bond lengths and angles for compound **4b**.

Bond	Bond length *A^o^*	Bond	Bond angle
O2-C14	1.224 (4)	N7-N3-C14	122.5 (3)
N3-N7	1.390 (3)	N3-N7-C10	116.6 (3)
N3-C14	1.357 (4)	N7-C10-C8	114.6 (4)
C6-C14	1.447 (5)		
N7-C10	1.306 (4)		

**Scheme 2:**
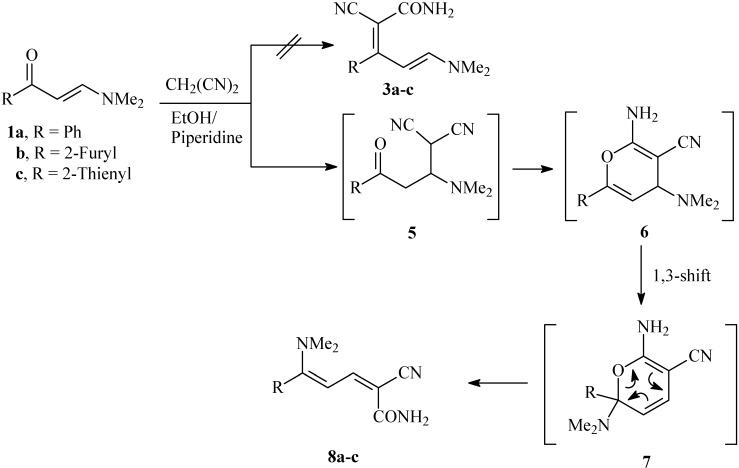
Proposed mechanism for the formation of 2-cyano-5-(dimethylamino)-5-aryl-penta-2,4-dienamides **8a-c**.

Disconnection of **4a-c** and consideration of the reported data has led us to believe that the products initially thought to be **3a-c** are in fact **8a-c**, which are formed *via* initial 1,4-addition of malononitrile across the double bond to yield **5** that cyclized to **6** then rearranged to **7**, that finally afforded **8*** via* anallowed 1,3-nitrogen shift (cf. Scheme 2). However, a possible conversion of **3 **to **8** involving migration of R *via* a 1,3 shift should not be overlooked. We wish to state that both **8** and **3** have the same molecular formulae and same spectral data, which after further inspection, established the structures **8a-c**. Thus, assuming that H-4 are shielded by nitrogen lone pair anisotropic effect, while H-3 are deshielded by electron attracting substituents; it is hence logic to assign the doublets at δ_H_ = ca. 5.68 ppm to H-4, while the doublets at δ_H_ = ca. 7.39 ppm would correspond to H-3. If the reaction products were **3a-c,** then H-4 in these assumed structures are shielded and H-5 are deshielded. We note that the deshielded doublet for **8a** at δ_H_ = ca. 7.39 ppm are correlated in the HMBC experiments with the amide carbonyl group at δ_C_ = ca. 164.90 ppm. If the reaction product was **3a**, such a correlation should not exist. Moreover, the methyl protons at δ_H_ = ca. 2.99 ppm show a cross peak correlation with C-5 at δ_C_ = ca. 157.93 ppm. This carbon was proven by DEPT experiments to be quaternary, consistent with structure **8a**. If, on the other hand, the reaction product were **3a**, then the methyl protons should be correlated with a carbon bearing a proton. 

**Scheme 3:**
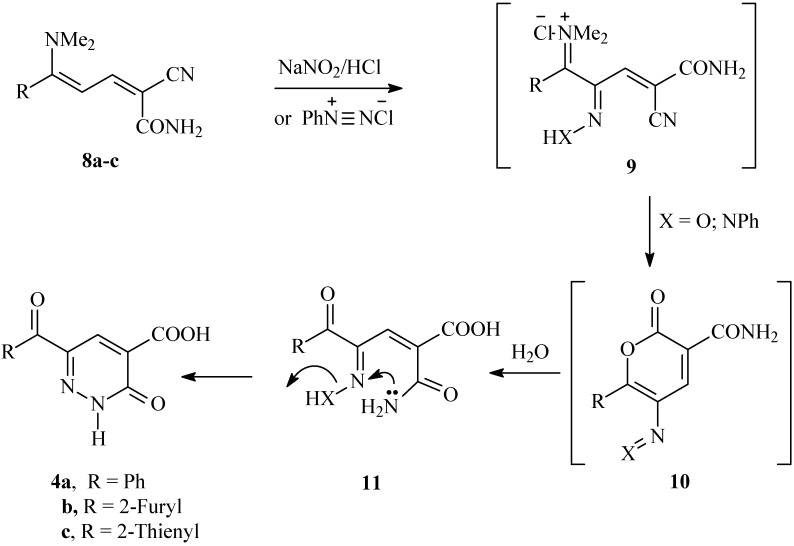
Proposed mechanism for the nitrozation and coupling reactions of 2-cyano-5-(dimethylamino)-5-phenylpenta-2,4-dienamides **8a-c**.

**Scheme 4:**
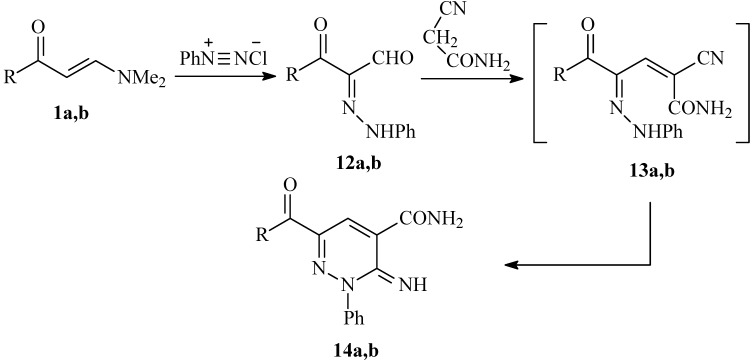
Reported synthesis of 2,3-dihydropyridazine-4-carboxamides **14a,b** from the condensation of arylhydrazonals **12a,b** with cyanoacetamide.

Consequently, a plausible mechanism for the formation of compounds **4a-c **is illustrated in Scheme 3. It is assumed that the initially formed **9 **is subject to an intramolecular cyclization to **10**, which is further hydrolysed into **11** under the reaction conditions. Finally, the lone pairs on the amide nitrogen then react with the oxime nitrogen or the hydrazone nitrogen kicking out either a water molecule or aniline, thus producing **4a-c. ** To our knowledge, this is the first reported cyclization *via* aniline elimination in such a system. It is worth mentioning that condensing 3-aroyl-2-(2-phenylhydrazono)propanals **12a,b **with cyanoacetamide has been reported to yield the 2,3-dihydropyridazine-4-carboxamides **14a,b **[15] (cf. Scheme 4). 

**Scheme 5:**
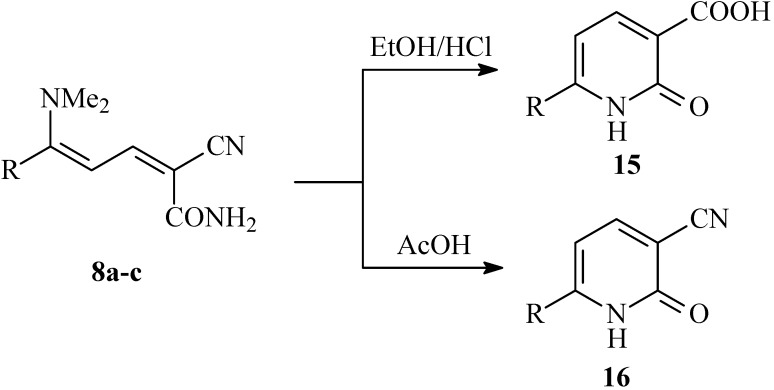
Synthesis of 1,2-dihydropyridine-3-carboxylic acids **15a-c **and 1,2-dihydropyridine-3-carbonitriles **16a-c**.

Conversions of **8a-c** into nicotinic acid derivatives **15a-c** were achieved by boiling in EtOH/HCl. When however compounds **8a-c** are heated under reflux in AcOH, nicotinic nitrile derivatives **16a-c **are obtained (cf. Scheme 5). 

**Scheme 6:**
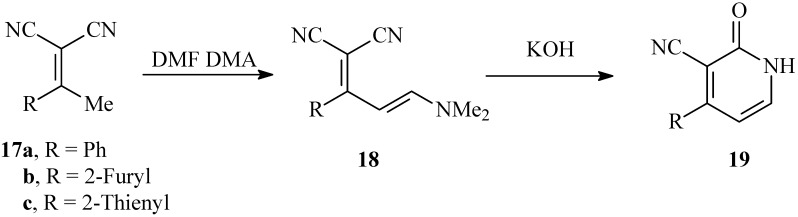
Reported synthesis of isomeric pyridones **13a-c**.

It has been reported earlier [16] that hydrolysis of **18** obtained *via* condensation of **17** with dimethylformamide dimethylacetal afforded the pyridone derivatives **19.** We have repeated this experiment and came to the conclusion that hydrolysis of **18 **in KOH affords in fact isomeric pyridone **19,** which in turn gives spectra very similar to those of **16.** Indeed, the mixed m.p. of the two products proves that they are different (cf. Scheme 6).

## Conclusions

We are now able to correct a previously reported initial 1,2-addition of malononitrile at the carbonyl moiety of enaminones **1a-c **and suggest instead the novel compounds **8a-c **as precursors for syntheses of pyridazinones and pyridones derivatives.

## Experimental

### General

Melting points were determined on a Shimadzu-Gallenkamp apparatus and are uncorrected. Elemental analyses were obtained by means of a LECO CHNS-932 Elemental Analyzer. NMR spectra were measured in DMSO-*d_6_* using a Bruker DPX 400 MHz superconducting spectrometer; HMQC, DEPT and NOE spectra were measured using Bruker Avance II 600 MHz superconducting spectrometer, and FT-IR measurements were from a Perkin Elmer 2000 FT-IR system. Mass spectrometric analysis was carried out on a VG-Autospec-Q high performance tri-sector GC/MS/MS.

### General procedure for the preparation of compounds ***8a-c***

A mixture of equimolecular amounts of each of enaminones **1a-c **(10 mmol) and malononitrile (10 mmol, 066 g) in EtOH (10 mL) was refluxed for 1 hr in the presence of few drops of piperidine. Upon cooling to r.t. a solid product precipitated, which was collected by filtration and crystallized from dioxane. 

*2-Cyano-5-(dimethylamino)-5-phenylpenta-2,4-dienamide* (**8a**): Yellow crystals, yield (72 %, 1.77 g); mp 257-258 ^o^C; IR (cm^-1^): 3435 and 3334 (NH_2_), 2195 (CN) and 1666 cm^-1^ (CO); MS m/z (M)^+^ = 241; ^1^H-NMR: d = 2.78 (s, 3H, NCH_3_), 3.14 (s, 3H, NCH_3_), 5.64 (d, 1H, *J* = 12.8 Hz, H-4), 6.82 (s, 2H, NH_2_), 7.13 (d, 1H, *J* = 12.8 Hz, H-3), 7.25-7.28 (m, 2H, phenyl-H), 7.34-7.65 (m, 3H, phenyl-H); ^13^C-NMR: d = 165.08 (CONH_2_), 164.59 (C-5), 153.27 (C-3), 133.99, 129.56, 128.76, 128.72, 118.90, 96.91, 87.57, 41.92 (N(CH_3_)_2_); Anal. calcd. for C_14_H_15_N_3_O: (241.12): C, 69.69; H, 6.27; N, 17.41. Found: C, 69.55; H, 6.07; N, 17.29.

*2-Cyano-5-(dimethylamino)-5-(furan-2-yl)penta-2,4-dienamide* (**8b**): Brownish red crystals, yield (75 %, 1.73 g); mp 245-246 ^o^C; IR (cm^-1^): 3331 and 3292 (NH_2_), 2190 (CN) and 1671 cm^-1^ (CO); MS m/z (M)^+^ = 231; ^1^H-NMR: d = 2.97 (s, 6H, N(CH_3_)_2_), 5.59 (d, 1H, *J* = 12.5 Hz, H-4), 6.72 (d, 1H, *J* = 5.0 Hz, furyl H-3), 6.78 (d, 1H, *J* = 5.0 Hz, furyl H-5), 7.02 (s, 2H, NH_2_), 7.56 (d, 1H, *J* = 12.5 Hz, H-3), 7.99 (t, 1H, *J* = 5.0 Hz, furyl H-4); ^13^C-NMR: d = 164.49 (CONH_2_), 153.40 (C-5), 151.73, 145.37, 144.80, 118.38, 115.75, 111.53, 98.31, 90.40, 40.77; Anal. calcd. for C_12_H_13_N_3_O_2_: (231.10): C, 62.33; H, 5.67; N, 18.17. Found: C, 61.97; H, 5.61; N, 18.21.

*2-Cyano-5-(dimethylamino)-5-(thiophen-2-yl)penta-2,4-dienamide* (**8c**): Yellow crystals, yield (72 %, 1.77 g); mp 258-259 ^o^C; IR (cm^-1^): 3403 and 3328 (NH_2_), 2196 (CN) and 1669 cm^-1^ (CO); MS m/z (M)^+^ = 247; ^1^H-NMR: d = 2.99 (s, 6H, N(CH_3_)_2_), 5.68 (d, 1H, *J* = 12.5 Hz, H-4), 6.94 (s, 2H, NH_2_), 7.18 (d, 1H, *J* = 5.0 Hz, thienyl H-3), 7.23 (t, 1H, *J* = 5.0 Hz, thienyl H-4), 7.39 (d, 1H, *J* = 12.5 Hz, H-3), 7.88 (d, 1H, *J* = 5.0 Hz, thienyl H-5); ^13^C-NMR: d = 164.90 (CONH_2_), 157.93 (C-5), 153.06 (C-3), 133.66, 131.21, 130.16, 128.14, 119.04 (CN), 99.29 (C-4), 89.99 (C-2), 41.46 (N(CH_3_)_2_); Anal. calcd. for C_12_H_13_N_3_OS: (247.08): C, 58.28; H, 5.30; N, 16.99; S, 12.97. Found: C, 58.23; H, 5.29; N, 16.85; S, 12.74.

### General procedure for the preparation of compounds ***4a-c***

*Procedure A:* To a solution of each of compound **8a-c** (10 mmol) in dioxane (15 mL) and HCl (2 mL), was added dropwise a prepared solution of NaNO_2_ (0.85 g, 10 mmol) and sodium acetate (15 mmol) in water (10 mL). The mixture was stirred for 1h. and allowed to warm up to r.t. During this time a precipitate is formed. The reaction mixture is then filtered off and recrystallized from dioxane.

*Procedure B:* Coupling reaction was carried out following procedure described earlier [17], which involves coupling each of compounds **8a-c** with phenyldiazonium chloride in dioxane /AcONa. 

*6-Benzoyl-3-oxo-2,3-dihydropyridazine-4-carboxylic acid* (**4a**): Greenish crystals, yield (78 %, 1.90 g); mp 186-187 ^o^C; IR (cm^-1^): 3420 (OH), 3240 (NH), 1737, 1678 and 1661 cm^-1^ (3 CO); MS m/z (M)^+^ = 244; ^1^H-NMR: d = 7.59-7.62 (t, 2H, *J* = 7.2 Hz, phenyl-H), 7.75 (t, 2H, *J* = 7.2 Hz, phenyl-H), 7.96 (br. s, 1H, OH, D_2_O exchangeable), 8.03 (s, 1H, pyridazinyl H-5), 8.05-8.06 (m, 2H, phenyl-H), 8.25 (br. s, 1H, NH, D_2_O exchangeable); ^13^C-NMR: d = 187.43 (ketone CO), 162.42 (carboxylic acid CO), 161.22 (ring CO), 154.14, 134.93, 134.46, 132.70, 129.10, 129.01, 128.72; Anal. calcd. for C_12_H_8_N_2_O_4_: (244.05): C, 59.02; H, 3.30; N, 11.47. Found: C, 58.95; H, 3.29; N, 11.52

*6-(Furan-2-carbonyl)-3-oxo-2,3-dihydropyridazine-4-carboxylic acid* (**4b**): Beige crystals, yield (75 %,1.75 g); mp 213-214 ^o^C; IR (cm^-1^): 3419 (OH), 3202 (NH), 1744, 1688 and 1657 cm^-1^ (3 CO); MS m/z (M)^+^ = 234; ^1^H-NMR: d = 6.86 (t, 1H, *J =* 4.5 Hz, furyl H-4), 7.77 (d, 1H, *J =* 4.3 Hz, furyl H-3), 7.96 (br. s, 1H, OH, D_2_O exchangeable), 8.03 (s, 1H, pyridazinyl H-5), 8.26 (br. s, 1H, NH, D_2_O exchangeable), 8.27 (d, 1H, *J =* 4.5 Hz, furyl H-5); Anal. calcd. for C_10_H_6_N_2_O_5_: (234.03): C, 51.29; H, 2.58; N, 11.96. Found: C, 51.34; H, 2.61; N, 12.03.

*3-Oxo-6-(thiophene-2-carbonyl)- 2,3-dihydropyridazine-4-carboxylic acid* (**4c**): Yellow crystals, yield (72 %, 1.80 g); mp 201-202 ^o^C; IR (cm^-1^): 3434 (OH), 3220 (NH), 1776, 1746 and 1693 cm^-1^ (3 CO); MS m/z (M)^+^ = 250; ^1^H-NMR: d = 7.37 (t, 1H, *J =* 4.3 Hz, thienyl H-4), 7.97 (br. s, 1H, OH, D_2_O exchangeable), 8.04 (s, 1H, pyridazinyl H-5), 8.21 (d, 1H, *J =* 4.3 Hz, thienyl H-3), 8.27 (m, 2H, thienyl H-5 and NH); Anal. calcd. for C_10_H_6_N_2_O_4_S: (250.00): C, 48.00; H, 2.42; N, 11.20; S, 12.81. Found: C, 48.05; H, 2.40; N, 11.01; S, 12.72. 

### General procedure for the preparation of compounds ***15a-c***

Each of compounds **8a-c** (10 mmol) was refluxed in an EtOH/HCl mixture (3:1, 10 mL) for 30 min. Upon cooling to r.t. a solid product precipitated that was collected by filtration and recrystallized from EtOH. 

*2-Oxo-6-phenyl-1,2-dihydropyridine-3-carboxylic acid* (**15a**): Beige crystals, yield (89 %, 1.91 g); mp 260-262 ^o^C; IR (cm^-1^): 3380 (NH), and 1725 cm^-1^ (CO); MS m/z (M)^+^ = 215; ^1^H-NMR: d = 6.84 (d, 1H, *J =* 8.8 Hz, pyridyl H-5), 7.50-7.627 (m, 3H, phenyl-H), 7.79-7.80 (m, 2H, phenyl-H), 8.20 (d, 1H, *J =* 8.8 Hz, pyridyl H- 4), 12.79 (s, 1H, NH); Anal. calcd. for C_12_H_9_NO_3_: (215.20): C, 66.97; H, 4.22; N, 6.51. Found: C, 67.03; H, 4.20; N, 6.56.

*6-(Furan-2-yl)-2-oxo-1,2-dihydropyridine-3-carboxylic acid* (**15b**): Brownish crystals, yield (90 %, 1.85 g); mp 268-269 ^o^C; IR cm^-1^: 3387 (NH), and 1730 cm^-1^ (CO); MS m/z (M)^+^ = 205; ^1^H-NMR: d = 6.81 (t, 1H, *J =* 4.3 Hz, furyl H-4), 6.98 (d, 1H, *J =* 7.6 Hz, pyridyl H-5), 7.34 (d, 1H, *J =* 4.3 Hz, furyl H-3), 7.84 (br. s, 1H, OH, D_2_O exchangeable), 7.94 (br. s, 1H, NH, D_2_O exchangeable), 8.07 (d, 1H, *J =* 4.5 Hz, furyl H-5), 8.41 (d, 1H, *J =* 7.6 Hz, pyridyl H-4); Anal. calcd. for C_10_H_7_NO_4_: (205.04): C, 58.54; H, 3.44; N, 6.83. Found: C, 58.31; H, 3.44; N, 7.05.

*2-Oxo-6-(thiophen-2-yl)-1,2-dihydropyridine-3-carboxylic acid* (**15c**): Brownish crystals, yield (93 %, 2.0 g); mp 266-268 ^o^C; IR (cm^-1^): 3398 (NH) and 1721 cm^-1^ (CO); MS m/z (M)^+^ = 221; ^1^H-NMR: d = 7.18 (d, 1H, *J =* 7.4 Hz, pyridyl H-5), 7.30 (t, 1H, *J =* 4.5 Hz, thienyl H-4), 7.82 (br. s, 1H, OH, D_2_O exchangeable), 7.94 (br. s, 1H, NH, D_2_O exchangeable), 7.95 (d, 1H, *J =* 4.5 Hz, thienyl H-3), 7.99 (d, 1H, *J =* 4.5 Hz, thienyl H-5), 8.39 (d, 1H, *J =* 7.4 Hz, pyridyl H-4). Anal. calcd. for C_10_H_7_NO_3_S: (221.01): C, 54.29; H, 3.19; N, 6.33; S, 14.49. Found: C, 54.14; H, 3.39; N, 6.63; S, 14.56.

### General procedure for the preparation of compounds ***16a-c***

Each of compounds **8a-c** (10 mmol) was refluxed in AcOH (10 mL) for 30 min. Upon cooling to r.t. a solid product precipitated that was collected by filtration and crystallized from AcOH. 

*2-Oxo-6-phenyl-1,2-dihydropyridine-3-carbonitrile* (**16a**): White crystals, yield (92 %, 1.80 g); mp 292-294 ^o^C; IR (cm^-1^): 3151 (NH), 2226 (CN) and 1660 cm^-1^ (CO); MS m/z (M)^+^ = 196; ^1^H-NMR: d = 6.74 (d, 1H, *J =* 8.8 Hz, pyridyl H-5), 7.51-7.57 (m, 3H, phenyl-H), 7.80-7.82 (m, 2H, phenyl-H), 8.22 (d, 1H, *J =* 8.8 Hz, pyridyl H- 4), 12.81 (s, 1H, NH); Anal. calcd. for C_12_H_8_N_2_O: (196.06): C, 73.46; H, 4.11; N, 14.28. Found: C, 73.43; H, 4.00; N, 14.20.

*6-(Furan-2-yl)-2-oxo-1,2-dihydropyridine-3-carbonitrile* (**16b**): Beige crystals, yield (94 %, 1.75 g); mp 298-300 ^o^C; IR (cm^-1^): 3163 (NH), 2230 (CN) and 1661 cm^-1^ (CO); MS m/z (M)^+^ = 186; ^1^H-NMR: d = 6.72 (d, 1H, *J =* 8.8 Hz, pyridyl H-5), 6.78 (t, 1H, *J =* 5.0 Hz, furyl H-4), 7.62 (d, 1H, *J =* 5.0 Hz, furyl H-3), 8.03 (d, 1H, *J =* 5.0 Hz, furyl H-5), 8.16 (d, 1H, *J =* 8.8 Hz, pyridyl H-4), 12.82 (s, 1H, NH). Anal. calcd. for C_10_H_6_N_2_O_2_: (186.04): C, 64.52; H, 3.25; N, 15.05. Found: C, 64.48; H, 3.14; N, 15.10.

*2-Oxo-6-(thiophen-2-yl)-1,2-dihydropyridine-3-carbonitrile* (**16c**): Yellowish crystals, yield (95 %, 1.92 g); mp 300-302 ^o^C; IR (cm^-1^): 3093 (NH), 2226 (CN) and 1651 cm^-1^ (CO); MS m/z (M)^+^ = 202; ^1^H-NMR: d = 6.70 (d, 1H, *J =* 8.6 Hz, pyridyl H-5), 7.25 (t, 1H, *J =* 4.5 Hz, thienyl H-4), 7.88 (d, 1H, *J =* 4.5 Hz, thienyl H-3), 8.01 (d, 1H, *J =* 4.5 Hz, thienyl H-5), 8.13 (d, 1H, *J =* 8.6 Hz, pyridyl H-4), 12.82 (s, 1H, NH); Anal. calcd. for C_10_H_6_N_2_OS: (202.02): C, 59.39; H, 2.99; N, 13.85; S, 15.86. Found: C, 59.35; H, 3.09; N, 13.90; S, 15.79.

## References

[1] Ferraz H. M. C., Goncalo E. R. S. (2007). Recent preparations and synthetic applications of enaminones. Quim. Nova.

[2] Lue P., Greenhill J.V. (1997). Enamines in heterocyclic synthesis. Adv. Heterocycl. Chem..

[3] Elassar A.Z.A., El-Khair A.A. (2003). Recent Developments in the chemistry of enaminones. Tetrahedron.

[4] Stanovnik B., Svete J. (2004). Synthesis of heterocycles from alkyl 3-(dimethylamino) propenoates and related enaminones. Chem. Rev..

[5] Yermolayev S. A., Gorobets N. Y., Lukinova E. V., Shishkin O. V., Shishkina S. V., Desenko S. M. (2008). An efficient synthesis of N1-substituted 2,5-dioxo-1,2,5,6,7,8-hexahydro-3-quinolinecarboxamide via enolate salts. Tetrahedron.

[6] Gorobets N.Y., Yousefi B.H., Belaj F., Kappe C.O. (2004). Rapid microwave-assisted solution phase synthesis of substituted 2-pyridone libraries. Tetrahedron.

[7] Al-Saleh B., Abdel-Khalik M. M., Al-Enzy A., Elnagdi M. H. (1999). Synthesis of new azoloazine derivatives: new routes to 1,2,4-triazolo[4,3-a]pyrimidines, pyrazolo[1,5-a]pyridines and pyrazolo[3,4-b]pyridinones. J. Chem. Res..

[8] Abdel-Khalik M. M., Elnagdi M. H., Agamy S. M. (2000). Studies with functionally substituted heteroaromatics: the chemistry of *n*-phenylhydrazonylalkylpyridinium salts and of phenylhydrazonylalkylbenzoazoles. Synthesis.

[9] Abdel-Khalik M. M., Agamy S. M., Elnagdi M. H. (2000). Studies with 2-Arylhydrazono-3-Oxopropanals: A novel route to 4-aroyl-2-aryl-1,2,3-triazoles, 3-substituted 4-arylazopyrazoles, 2-substituted glyoxalonitrile and 3-oxoalkanonitriles. Z. Naturforsch.

[10] Agamy S. M., Abdel-Khalik M. M., Mohamed M. H., Elnagdi M. H. (2001). Enaminones as building blocks in heterocyclic synthesis: a new one pot synthesis of polyfunctional substituted pyridines. Z. Naturforch..

[11] Al-Qalaf F., Abdelkhalik M. M., Al-Enezi A., Al-Ajmi J. R. (2008). Studies with functionally substituted enamines: synthesis of 2-aroyl-3-dimethylamino-2-propenenitrile and their reactivity toward nitrogen nucleophiles. Heterocycles.

[12] Al-Omran F., Al-Awadi N., El-Khair A. A., Elnagdi M. H. (1997). Synthesis of new aryl and heteroaromatic substituted pyridines, pyrazoles, pyrimidines and pyrazolo[3,4-*d*]pyridazine. Org. Prep. Proced. Int..

[13] Al-Omran F., Al-Awadi N., Abdel-Khalik M. M., Kaul K., El-Khair A. A., Elnagdi M. H. (1997). Substituted 3-dimethylaminoprop-2-en-1-ones as building blocks in heterocyclic synthesis : routes to 6-aryl and 6-heteroaryl-2*H*-pyran-2-ones and 6- and 4-aryl-1,2-dihydropyridine-2(1*H*)-ones. J. Chem. Res..

[B14-molecules-14-00068] 14.CCDC 705064 contains the supplementary crystallographic data for this paper. These data can be obtained free of charge from The Cambridge Crystallographic Data Center via www.ccdc.cam.ac.uk/data_request/cif.

[15] Agamy S. M. (2001). 1-Substituted 3-dimethylaminoprop-2-en-1-ones as building blocks in heterocyclic synthesis: new routes to 6-aroylpyridazin-3-ones, 4,6-diaroylpyridazin-3-imines and 3-aroylpyrazolo[5,1-c][1,2,4]triazines. J. Chem. Res..

[16] Al-Omran F., Abdel-Khalik M. M., Elnagdi M. H. (1995). Studies with polyfunctionally substituted heteroaromatics: new routes for the synthesis of polyfunctionally substituted pyridines and 1,2,4-triazolo[1,5-a]pyridines. J. Heteroatom Chem..

[17] Al-Omran F., Abdel-Khalik M. M., El-Khair A. A., Elnagdi M. H. (1997). Studies with functionally substituted heteroaromatics: a novel route for the synthesis of 1-aryl-6-oxopyridazinones, 1-arylpyridazine-6-imines and 1-aryl-6-imino-4-pyridazinals. Synthesis.

